# The Effects of Dietary Loading on Chondrocytes Transdifferentiation During Postnatal Maxillary Growth

**DOI:** 10.1111/ocr.70111

**Published:** 2026-03-11

**Authors:** Michael Stark, Paria Dehghanian, Ying Liu, LouisBruno Ruest, Matthew J. Kesterke, Yan Jing

**Affiliations:** ^1^ Department of Orthodontics Texas A&M College of Dentistry Dallas Texas USA; ^2^ Department of Biomedical Sciences Texas A&M College of Dentistry Dallas Texas USA

**Keywords:** cell transdifferentiation, dietary loading, mid‐palatal suture, orthodontics

## Abstract

**Background:**

The mid‐palatal suture is a key growth site for transverse maxillary expansion, yet the cellular mechanisms governing its postnatal development and response to mechanical loading remain poorly defined. This study investigated the contribution of chondrocyte‐derived osteogenesis to maxillary bone formation and the influence of dietary loading on this process.

**Methods:**

Two chondrocyte‐lineage tracing mouse models were utilised: Aggrecan‐Cre^ERT2^ (Acan^Lineage^); R26R^TdTomato^; 2.3Col1a1‐GFP and Col10a1‐Cre (Col10a1^Lineage^); R26R^TdTomato^; 2.3Col1a1‐GFP. Twelve 3‐week‐old male mice per genotype were randomly assigned to either a hard diet (control) or soft diet (experimental) for 6 weeks. A single tamoxifen injection was administered to Acan^Lineage^ mice at 3 weeks to induce Cre event. Radiographic, uCT and histomorphometric analyses were performed.

**Results:**

Soft diet feeding significantly reduced maxillary arch width, bone volume and bone mineral density in the mid‐palatal region. Although chondrocyte proliferation was comparable between groups, differentiation was notably impaired. Lineage tracing demonstrated that both Acan^Lineage^‐ and Col10a1^Lineage^‐derived bone cells were more abundant under hard diet conditions, while non‐chondrocyte‐derived bone cell numbers remained similar between groups. The population of Acan^Lineage^‐derived pre‐osteoblasts and osteocytes was markedly lower in soft diet‐fed mice.

**Conclusions:**

Acan^+^ chondrocytes in the mid‐palatal suture contribute to postnatal maxillary bone formation through transdifferentiation. Dietary mechanical loading regulates both chondrogenesis and chondrocyte transdifferentiation, ultimately affecting maxillary bone growth and remodelling.

## 1 Background

Understanding craniofacial growth and maturation is essential for orthodontists to provide effective treatment to patients. While multiple genetic and environmental factors affect facial growth, abundant evidence shows that masticatory function has a significant influence on the development of the craniofacial complex. For example, bite forces provide tensile strains sufficient to separate the skeletal sutures, leading to function‐induced bone modelling and remodelling of the sutures [1]. However, the regulatory mechanisms of masticatory stimuli on suture growth are not fully understood.

Maxillary constriction is of particular importance to orthodontists. It is associated with increased dental crowding, decreased nasal air volume [2], and excessive vertical growth. Both clinical and animal studies have shown that decreased masticatory forces due to a softer diet result in narrower maxillary arches [3], whereas rapid palatal expansion (RPE) corrects jaw width, crowding and cross bites [4].

Maxillary transverse growth adapts to mechanical stimulation of the mid‐palatal suture, which is the primary contributor to transverse maxillary growth [5] and the main site of resistance to RPE [6]. It is a fibrocartilaginous joint composed of bone and a layer of secondary cartilage in the middle of the suture [7]. Secondary cartilage is particularly responsive to external stimuli and matures through endochondral ossification, which begins with mesenchymal cells that proliferate and differentiate into chondrocytes. As the cartilage grows, chondrocytes become entrapped and enter a hypertrophic state.

It was traditionally believed that hypertrophic chondrocytes undergo apoptosis, after which cells from the underlying bone marrow invade the calcified cartilage, initiating angiogenesis and subsequent bone deposition [8]. However, recent cell lineage tracing studies, including our own work [9], have challenged this classical view. We and others [10] have demonstrated that terminal hypertrophic chondrocytes can directly transdifferentiate into osteocytes without reverting to a stem cell stage, in regions such as the temporomandibular joint (TMJ) condylar ramus and long bones. This chondrocyte‐formed bone (CFB) constitutes approximately 70%–80% of endochondral bone. Collectively, these findings establish that chondrocytes play a pivotal role in endochondral ossification and that chondrogenesis and osteogenesis represent a continuous, integrated process. However, it still remains unclear whether chondrocytes within the mid‐palatal suture directly contribute to postnatal maxillary growth.

This study was designed to clarify how chondrocytes within the mid‐palatal suture contribute to maxillary growth during late postnatal development and to determine whether mechanical stimulation from dietary loading influences this process. We proposed that diminished mechanical input would disrupt chondrocyte‐derived osteogenesis, leading to impaired maxillary transverse expansion. Demonstrating that chondrocytes actively sense and respond to mechanical cues during suture growth would not only redefine the cellular mechanisms of maxillary development but also open new avenues for biomechanical and regenerative approaches to correct maxillary transverse deficiencies.

## 2 Methods

### 2.1 Animals

Two Cre mouse lines were used to trace chondrocyte lineage. The Aggrecan(Acan)‐Cre^ERT2^ line was employed to label all chondrocytes and their offspring following tamoxifen induction at 3 weeks of age, while the Col10a1‐Cre line was used for constitutive labelling of hypertrophic chondrocytes and their descendants, beginning at approximately embryonic day 14.5 (E14.5) [11].

Male Acan^Lineage^ (Aggrecan‐Cre^ERT2^; R26R^TdTomato^; 2.3Col1a1‐GFP) and Col10a1^Lineage^ (Col10a1‐Cre; R26R^TdTomato^; 2.3Col1a1‐GFP) mice were used (*n* = 12 per genotype). At 3 weeks of age, animals were randomly assigned to one of two dietary conditions: a hard diet group fed standard pellets (*n* = 6) or a soft diet group (*n* = 6) fed powdered chow of identical composition (Figure S1A). Mice were maintained on their respective diets for 6 weeks. The 3‐week developmental stage in mice corresponds to the prepubescent period in humans (approximately 6–8 years of age) [12].

At 9 weeks of age, all mice were sacrificed for analysis. To label proliferating cells, EdU (10 μg/mL; Invitrogen, A10044) was administered 3 h before tissue harvest. Sample size was based on published estimates assuming a power of 0.95 and alpha of 0.05, with a 10% difference and effect size of 2.4 [13].

### 2.2 Radiographs

**FIGURE 1:**
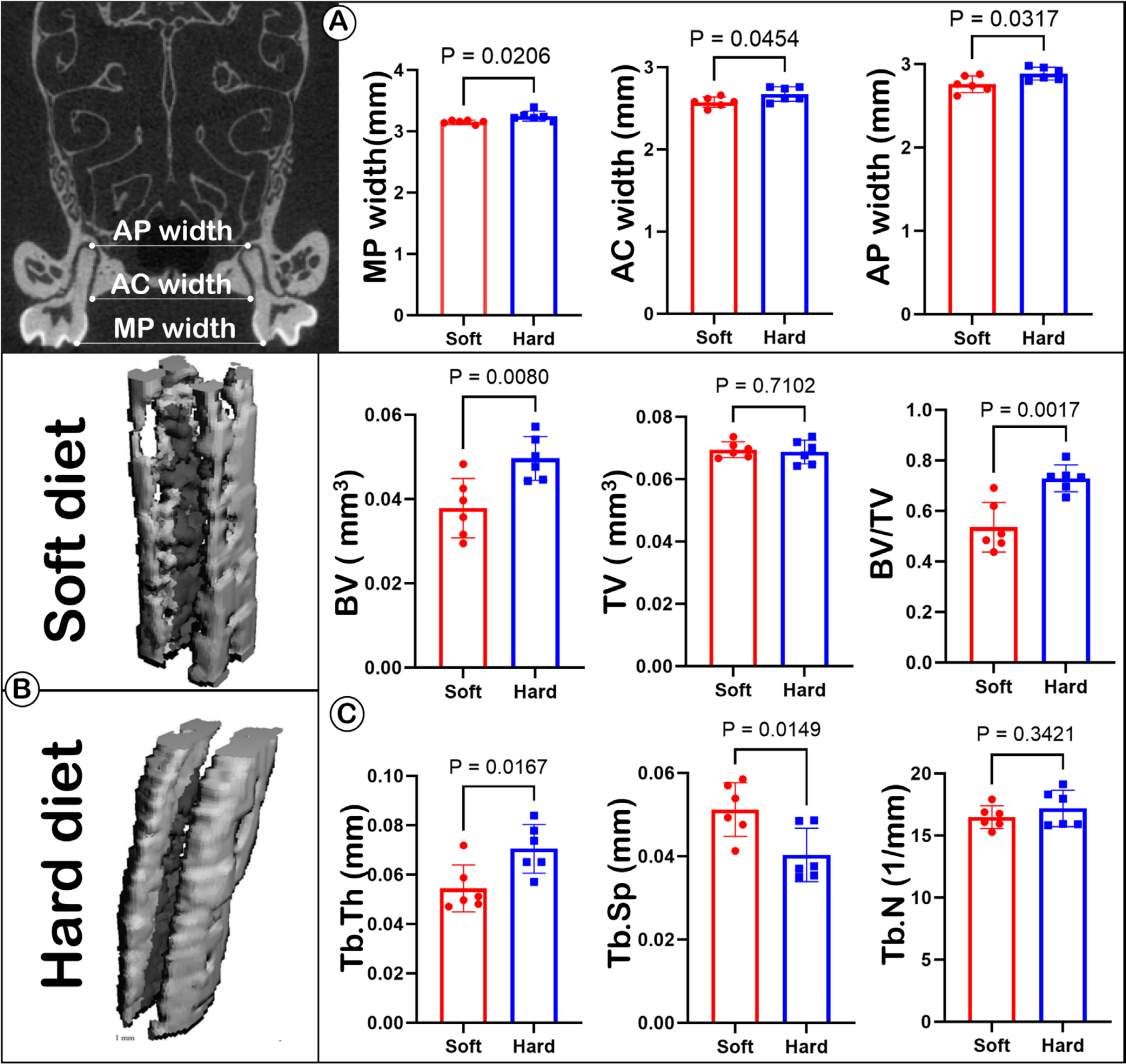
Radiological analysis for control and soft diet groups. (A) Maxillary width was measured at three anatomical levels, all of which showed a significant reduction in the soft diet group compared to controls. (B) Representative 3D reconstructed images of the maxillary bone within the region of interest (ROI). (C) Quantitative μCT analysis confirmed significant decreases in bone parameters in the soft diet group.

The maxillae were fixed in 4% paraformaldehyde and dissected. μCTs (μCT 35, Scanco Medical, Switzerland) were taken to quantify bone quality between the palatal roots of the 1st and 2nd molars 100 μm adjacent to the mid‐palatal suture (Figure S1B). The μCT 3D reconstruction of the maxillary bone was oriented so that the occlusal plane was perpendicular to the mid‐palatal suture. 2D images were captured using that orientation to measure the maxillary width at three levels (Figure 1A): MP (mesial‐palatal cusp of the 1st molar), AC (alveolar crest at the 1st molar) and AP (apex of the palatal root of the 1st molar).

### 2.3 Cell Proliferation, Immunohistochemistry and Confocal Microscopy

The maxilla was decalcified in 10% EDTA, dehydrated in 30% sucrose, and sectioned on the transverse plane along the axis of the mid‐palatal suture. The sections for analysis were selected by one examiner where the transverse width of the mid‐palatal suture between the maxillary 1st and 2nd molars was largest. The region of interest (ROI) for chondrogenic activity was the mid‐palatal suture between the palatal roots of the maxillary 1st and 2nd molars; the ROI for osteogenic activity was defined as the bone region located 100 μm lateral to the suture ROI on both sides (Figure S1B). The sections were used for Toluidine blue staining and immunohistochemistry [14] (IHC) with the following antibodies: Aggrecan (the major proteoglycan in the articular cartilage [15], Abcam; 1:400), Col10a1 (Collagen type 10a1 expressed by hypertrophic chondrocytes [15], Abcam; 1:400), Osx (a key transcriptional factor for osteoblast differentiation [16], Abcam; 1:100), DMP1 (a marker expressed by osteocytes [17], generously provided by Dr. Chunlin Qin; 1:100). Click‐iT Imaging Kit with Alexa Fluor 647‐azide (A10277) was used to detect EdU signal in cryosections for cell proliferation analysis [18]. The fluorescent images were captured using an SP8 Leica confocal microscope at wavelengths ranging from 488 (green) to 633 (red) nm [17].

Chondrogenic activity was evaluated between the palatal roots of the 1st and 2nd molars (Figure S1B, left) based on the following parameters: (1) the density of Acan^+^ (Tomato^+^) chondrocytes (Acan^+^ cells/cartilage area); (2) the density of EdU^+^ chondrocytes (EdU^+^ cells/cartilage area); and (3) the expression levels of Aggrecan and Col10a1 (marker^+^ area/cartilage area) [19].

Chondrocyte transdifferentiation was evaluated in the bone adjacent to the mid‐palatal suture (100 mm away from the suture bilaterally (Figure S1B) by merging Tomato and GFP signals with the following parameters [19]): (1) the numbers of Acan^Lineage^ or Col10a1^Lineage^‐derived bone cells (in yellow and red colour); (2) the number of non‐chondrocyte‐derived bone cells (in green and blue colour); and (3) the ratio of Acan^Lineage^ or Col10a1^Lineage^‐derived bone cells to all subchondral bone cells to represent the percentage of chondrocyte‐derived osteogenesis.

In addition, the Acan^Lineage^ tracing was co‐localised with Osx [16] or DMP1 [17] IHC to reflect early or mature osteogenic cells, respectively [14]. The numbers of Acan^Lineage^‐derived Osx^+^ or DMP1^+^ cells, the numbers of non‐Acan^Lineage^‐Osx^+^ or DMP1^+^ cells, and the percentage of Acan^Lineage^‐Osx^+^ or DMP1^+^ cells over the total number of Osx^+^ or DMP1^+^ cells were measured to reflect the dietary effects on chondrocyte transdifferentiation at different osteogenic stages.

### 2.4 Statistics

All statistical analyses were conducted using SPSS (Version 28.0, IBM). Based on the skewness and kurtosis statistics, the data were normally distributed. For quantitative analysis on the histological level, four sections were averaged for each animal. Parametric means and standard deviations were used to describe central tendencies and dispersion. Mann–Whitney *U* tests were conducted for all statistical comparisons. Intraobserver error was assessed using intraclass correlation coefficients (ICC) of repeated measures, with all measures having ICC > 0.99.

## 3 Results

### 3.1 Weights

There was no statistical significance in weight between groups prior to diet modification (Figure S1C). During weeks four through six, mice from the soft food diet group weighed significantly less than the control group (Figure S1C). However, there was no statistically significant difference between groups in total weight change from the start to the end of the experiment (Figure 1D).

### 3.2 Soft Diet Demonstrated Narrower Maxillary Measurements and Lower Bone Quality

The soft food mice had significantly narrower maxillary widths measured at MP, AC and AP than the control mice (Figure 1A). The 3D μCT image showed the palatal bone adjacent to the mid‐palatal suture was more porous in the soft food diet mice than in the control group (Figure 1B). Quantitatively, both bone volume (BV) and the bone volume to total volume ratio (BV/TV) were significantly reduced in the soft food diet group, with significantly thinner trabeculae but larger trabecular spaces. There was no significant between‐group difference in trabecular number (Figure 1C). In summary, the radiological analysis revealed narrower maxillary arches and reduced bone among mice fed softer diets.

### 3.3 The Chondrogenic Activity Decreased in the Mid‐Palatal Suture From Soft Diet Mice

**FIGURE 2:**
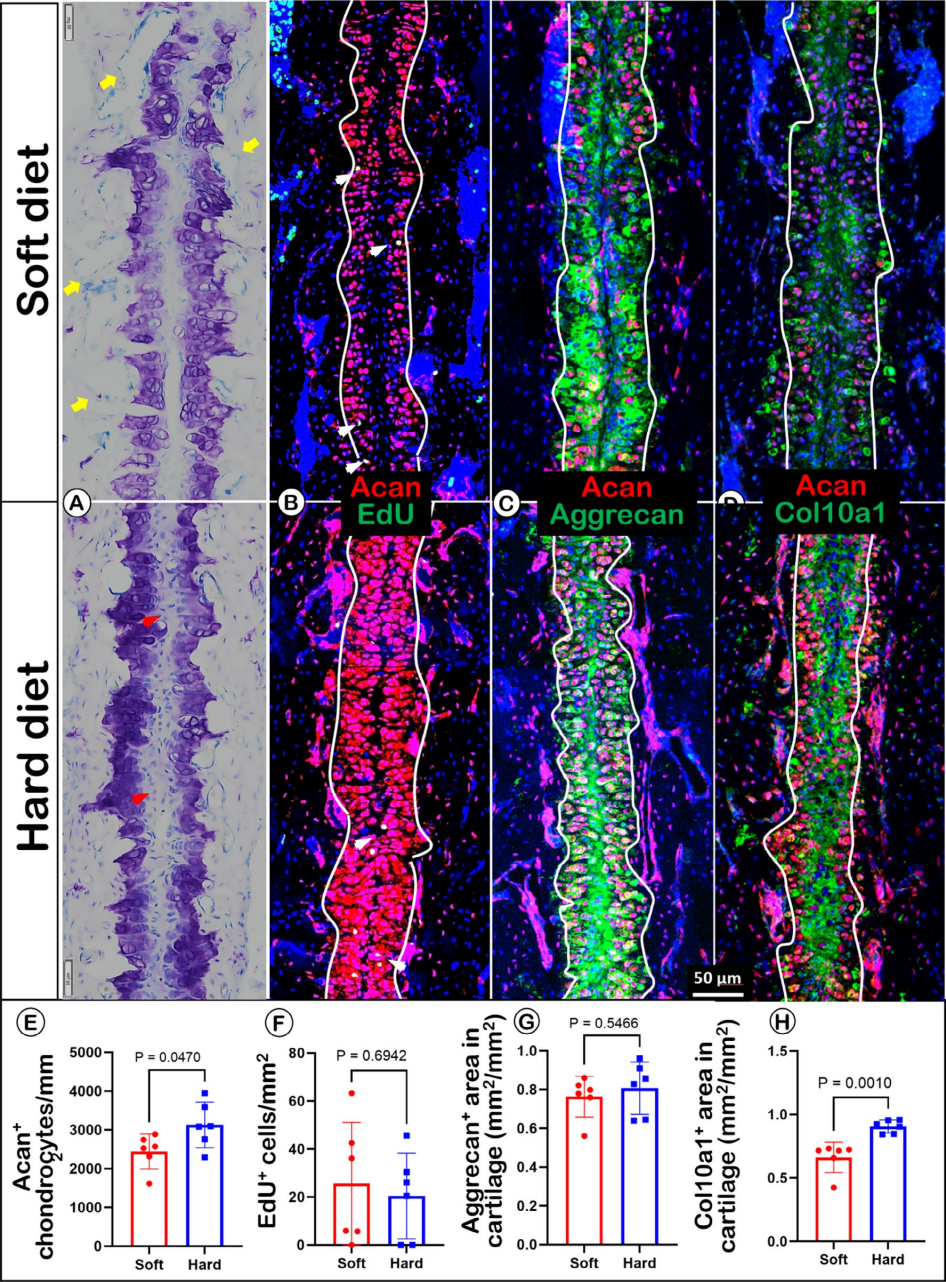
Reduced dietary loading suppresses chondrogenic activity in the mid‐palatal suture. (A) Toluidine blue staining revealed diminished cartilaginous matrix and increased bone marrow spaces (yellow arrows) in the soft diet group, with notably fewer chondrogenic progenitors in the central region of the suture (red arrows). (B) Confocal imaging of EdU labelling showed few proliferating cells (arrows) in both diet groups; however, the number of Acan^+^ chondrocytes was markedly reduced in the soft diet group. (C) IHC for Aggrecan (green) showed comparable expression between groups. (D) In contrast, Col10a1 expression (green) was substantially decreased in the soft diet group, indicating impaired chondrocyte maturation. (E–H) Quantitative analyses of Acan^+^ cell density (E), EdU^+^ cell density (F), and Aggrecan (G) and Col10a1 (H) expression levels were consistent with the qualitative observations. White lines delineate the interface between the mid‐palatal cartilage and adjacent trabecular bone.

Toluidine blue staining revealed reduced purple intensity in the cartilage and more marrow spaces in the bone adjacent to the mid‐palatal suture in the soft diet group (Figure 2A), indicating a lower abundance of cartilaginous matrix and bone. Additionally, numerous chondrogenic progenitors were observed in the central region of the control group's cartilage, whereas these cells were scarcely present in the soft diet group (Figure 2A, red arrows).

The confocal images showed few EdU^+^ positive cells in the mid‐palatal sutures of both groups, but there were far fewer red Acan^+^ chondrocytes in the soft diet group than in the controls (Figure 2B). The quantitative results confirmed a significant decrease in the density of Acan^+^ chondrocytes (Figure 2E) with no significant between‐group difference in cell proliferation (Figure 2F). The level of Aggrecan expression in the mid‐palatal suture was similar between groups (Figure 2C), with no significant difference in the ratio of Aggrecan^+^ area to cartilage area (Figure 2G). However, Col10a1 expression was much lower in the soft diet group (Figure 2D), with the ratio of Col10a1^+^ area to cartilage area being significantly decreased than that of the controls (Figure 2H). In summary, these results showed that the soft diet had a mild effect on chondrocyte proliferation but a significant impact on chondrocyte differentiation and maturation in the mid‐palatal suture.

### 3.4 Soft Diet Reduces the Transdifferentiation of Chondrocytes Into Bone Cells

**FIGURE 3:**
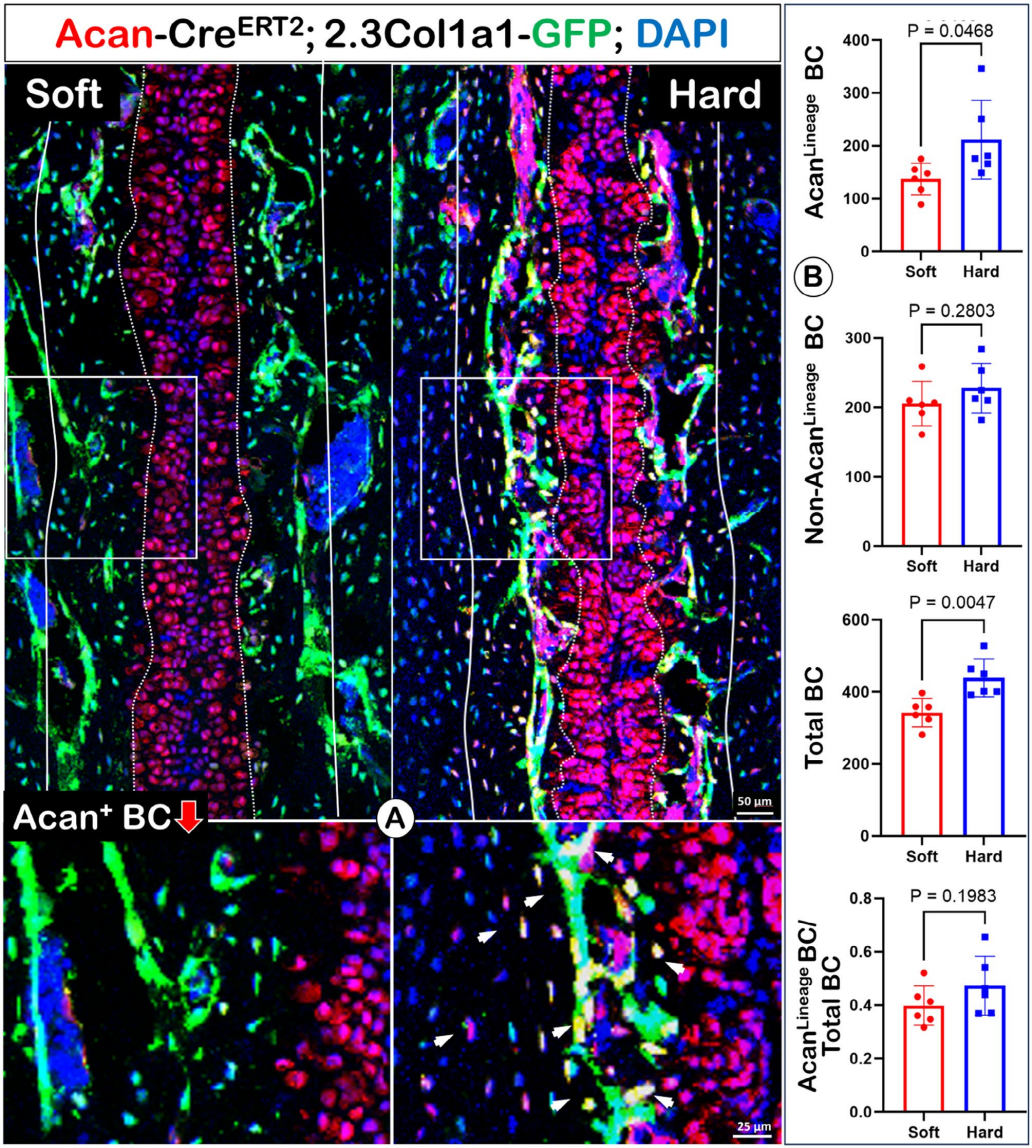
Reduced dietary loading diminishes chondrocyte‐derived osteogenesis in Acan^Lineage^ mice. (A) Confocal images of the region of interest (ROI) revealed abundant Acan^Lineage^‐derived bone cells (yellow or red) in the hard diet group, which were markedly reduced in the soft diet mice. (B) Quantitative analysis showed significant decreases in the numbers of Acan^Lineage^‐derived bone cells, total bone cells, and the ratio of Acan^Lineage^‐derived to total bone cells in the soft diet group, whereas the number of non‐Acan^Lineage^‐derived bone cells (green or blue) remained comparable between groups. BC: Bone cells. White dotted lines indicate the boundary between the mid‐palatal cartilage and adjacent trabecular bone; white solid lines represent an area approximately 100 μm lateral to the dotted boundary.

Confocal imaging of both Acan^Lineage^ (Acan‐Cre^ERT2^; Figure 3A) and Col10a1^Lineage^ (Col10a1‐Cre; Figure S2A) mice revealed comparable qualitative patterns. In hard diet Acan^Lineage^ mice, a higher density of red and yellow chondrocyte‐derived bone cells was observed surrounding the suture compared to soft diet mice. Quantitative analyses corroborated these observations. Both the total number of Acan^Lineage^‐derived bone cells and the overall bone cell count were significantly greater in hard diet mice than in soft diet mice. In contrast, the number of non–Acan^Lineage^‐derived bone cells did not differ significantly between dietary groups, resulting in a similar ratio of Acan^Lineage^‐derived to total bone cells between groups (Figure 3B). The Col10a1^Lineage^ mice exhibited the same trend (Figure S2B).

Collectively, these data indicate that chondrocytes within the mid‐palatal suture actively transdifferentiate into bone cells, contributing substantially to maxillary bone formation during late postnatal growth. Prolonged reduction in dietary loading via a soft diet markedly suppressed this chondrocyte‐to‐bone conversion, leading to an overall reduction in bone cell number within the palatal region, while non‐chondrocyte‐derived bone cells remained unaffected.

**FIGURE 4:**
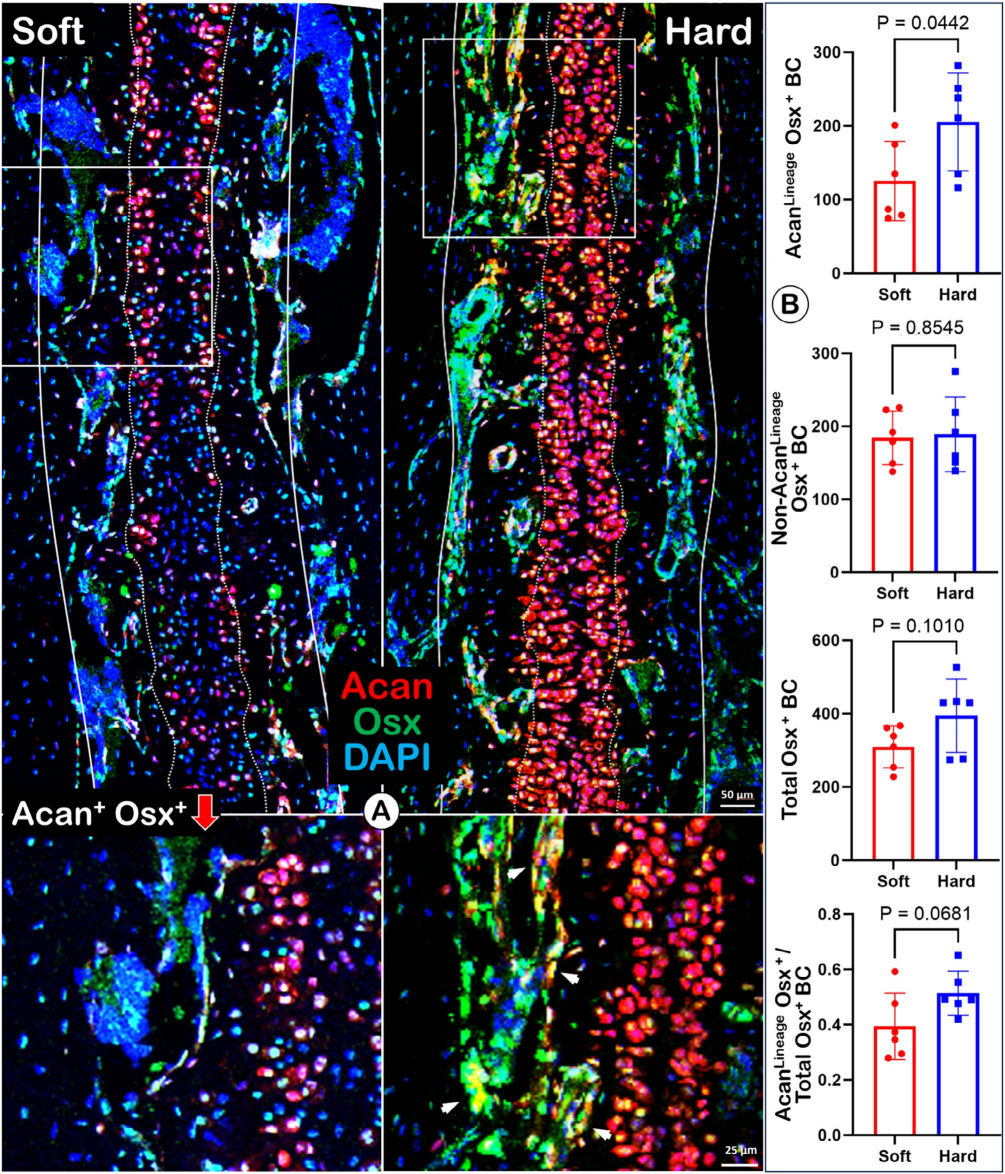
Decreased dietary loading disrupts chondrocyte transdifferentiation to early osteogenic cells. (A) In the hard diet group, confocal imaging revealed numerous Acan^+^ Osx^+^ cells (arrows) populating the subchondral bone region adjacent to the mid‐palatal suture, whereas the soft diet group exhibited a striking loss of these cells. (B) Quantitative analysis confirmed these observations. BC, Bone cells. White dotted lines outline the interface between mid‐palatal cartilage and trabecular bone; white solid lines indicate regions approximately 100 μm lateral to the boundary.

To further confirm the impact of dietary loading on chondrocyte transdifferentiation during maxillary bone development, Acan^Lineage^ was colocalized with IHC staining of Osx and DMP1, which reflect the early and late stages of osteogenic cells, respectively. The confocal images of Osx IHC showed numerous red bone cells with yellow nuclei in the control mice, indicating cell transdifferentiation from chondrocytes to Osx^+^ cells (Figure 4A, right). However, there were fewer yellow Acan^Lineage^‐Osx^+^ cells in the soft diet group (Figure 4A, left). The quantitative results confirmed significantly fewer Acan^Lineage^‐Osx^+^ bone cells in the soft diet than the control mice, with similar numbers of non‐Acan^Lineage^‐Osx^+^ bone cells (Figure 4B). The number of total Osx^+^ bone cells was also decreased in the soft diet group, but the group difference was not statistically significant, resulting in a lower ratio of Acan^Lineage^‐Osx^+^ bone cells to total Osx^+^ bone cells that approached a significant difference between groups (Figure 4B).

**FIGURE 5:**
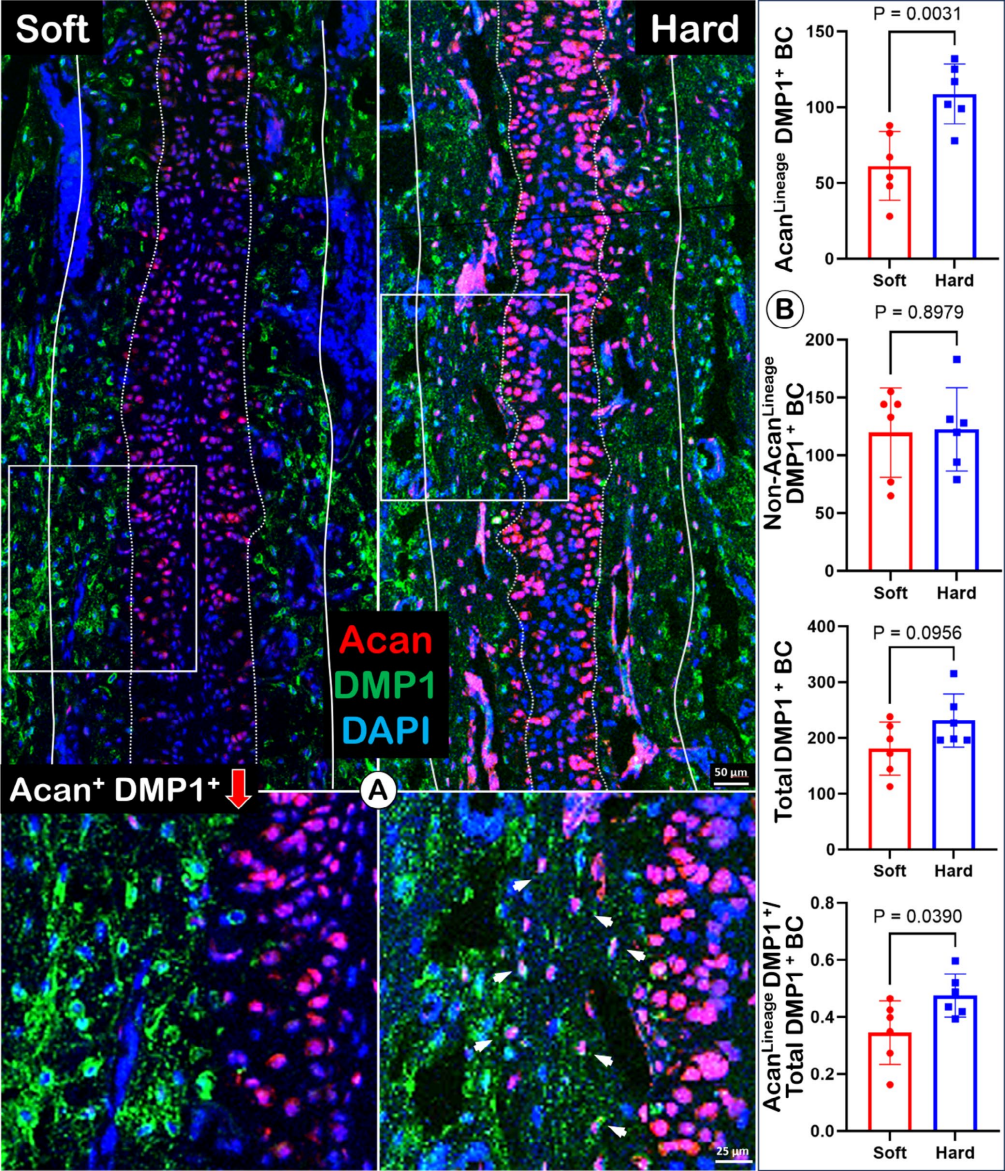
Reduced mechanical loading limits chondrocyte‐to‐osteocyte transition in the mid‐palatal suture. (A) In hard diet mice, confocal microscopy showed numerous Acan^+^ DMP1^+^ cells (arrows) integrated within the bone matrix surrounding the mid‐palatal suture, whereas soft diet mice displayed a marked depletion of these dual‐positive cells. (B) Quantitative evaluation corroborated these findings. BC, Bone cells. White dotted lines indicate the interface between cartilage and trabecular bone; white solid lines outline a region approximately 100 μm lateral to the suture margin.

Similarly, the confocal images showed abundant Acan^Lineage^‐DMP1^+^ osteocytes in the control mice but only a few in the soft diet mice (Figure 5A). The quantitative data showed significantly fewer Acan^Lineage^‐DMP1^+^ osteocytes in the soft diet group than in the hard diet group, but there were no significant differences in the number of non‐Acan^Lineage^‐DMP1^+^ osteocytes between groups (Figure 5B). There were also no significant between‐group differences in total DMP1^+^ cells, although there were fewer cells in soft diet than in the control group. This resulted in a significantly lower ratio of Acan^Lineage^‐DMP1^+^ cells to total DMP1^+^ cells in the soft diet group (Figure 5B).

## 4 Discussion

Soft diet is a well‐established experimental model for simulating reduced masticatory muscle loading in craniofacial growth studies. The present study found that consistent decreases in dietary loading for 6 weeks produced a narrower maxillary arch along with less bone volume, lower bone density, thinner trabecular thickness and larger trabecular spaces in the bone adjacent to the mid‐palatal suture. These results are consistent with previous studies [3]. Furthermore, using advanced lineage‐tracing tools we discovered that chondrocytes transdifferentiate into bone cells during adolescent maxillary growth, which demonstrates that chondrocytes are not passive structural cells but active progenitors of bone during maxillary expansion. Dietary loading is essential for sustaining this chondrocyte‐derived osteogenesis, and their absence results in reduced maxillary transverse growth and decreased bone quality.

Secondary cartilage is known to be remodelled in response to changes in muscle loading. The present study showed low chondrocyte proliferation in both control and soft diet groups with no significant difference between groups. In addition, the expression level of Aggrecan was also similar in both groups. However, the expression of Col10a1 was significantly impacted in the soft diet group, indicating diminished chondrocyte maturation. The decreased density of Acan^+^ chondrocytes traced after tamoxifen induction at 3 weeks further confirmed that reduced loading limits chondrocyte differentiation. These findings reveal that while proliferation remains stable, mechanical unloading primarily disrupts chondrocyte maturation and lineage progression within secondary cartilage.

Although the chondrogenic and osteogenic changes in the mid‐palatal suture in response to dietary loading have been well documented separately, no study had previously associated these changes together. The lineage tracing data in the present study bridges this gap by demonstrating that chondrocyte transdifferentiation is a continuous and diet‐sensitive process during late postnatal maxillary growth. In response to a hard diet, nearly half of the osteogenic population in the bone adjacent to the mid‐palatal suture originated from chondrocyte lineages (47% from Acan^Lineage^ and 33% from Col10a1^Lineage^). This direct lineage contribution highlights chondrocyte transdifferentiation as an essential cellular mechanism coupling mechanical loading to osteogenesis in the growing maxilla.

There were 35% and 32% fewer transdifferentiated bone cells in Acan^Lineage^ and Col10a1^Lineage^ soft diet mice, respectively, both of which were significantly different between groups. Furthermore, there were significantly fewer total bone cells in the bone around the suture of soft diet Acan^Lineage^ and Col10a1^Lineage^ mice (22% and 19%, respectively). These decreases were primarily driven by the loss of chondrocyte‐derived bone cells, as the number of non‐chondrocyte‐derived bone cells remained unchanged between groups. Consistent findings from both Acan‐Cre^ERT2^ and Col10a1‐Cre models confirm that dietary loading exerts a strong regulatory effect on chondrocyte transdifferentiation and subsequent bone formation.

**FIGURE 6:**
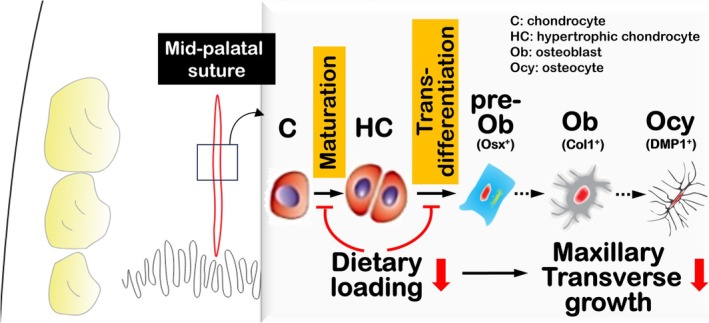
The diagram for working hypothesis. Under normal mechanical loading, chondrocytes within the mid‐palatal suture differentiate and transdifferentiate into bone‐forming cells, driving maxillary transverse expansion and maintaining bone integrity. When dietary loading is reduced, chondrocyte transdifferentiation is disrupted, resulting in parallel decreased chondrocyte‐derived osteogenic cells from early to late stages, and ultimately narrower maxillary width with poorer bone quality. This highlights that diminished chondrocyte transdifferentiation is a key mechanism underlying the reduction in maxillary bone formation and quality under soft diet conditions.

On the other hand, the soft diet mice showed 39% and 44% fewer Acan^Lineage^‐Osx^+^ (Figure 4) and DMP1^+^ (Figure 5) cells, respectively, but only 3% and 2% fewer non‐Acan^Lineage^‐Osx^+^ and DMP1^+^ cells, respectively. The ratio of Osx^+^ and DMP1^+^ cells derived from Acan^Lineage^ decreased 23.3% and 22.6% in soft diet mice, respectively. Gene expressions of *Osx*, *2.3Col1a1*‐GFP and *DMP1* reflect the maturation of osteogenic cells from the early, to middle, and late stages. Thus, the significant decrease in the number of Acan^+^ Osx^+^ cells demonstrated the direct regulation of dietary loading on chondrocyte cell fate change. In addition, the percent differences between groups in the ratio of chondrocyte‐derived bone cells from these three stages are not only similar to each other (19%–23.3%), but also to the percent differences in chondrocyte differentiation (22% less in Acan^+^ chondrocytes and 27% less in Col10a1 expression), highlighting the continuous nature of this chondrocyte‐to‐bone cell pathway (Figure 6). Together, these data establish that impaired chondrocyte‐derived osteogenesis is the principal mechanism underlying reduced bone formation in low dietary loading conditions.

The present study demonstrated that a softer diet led to a 5% reduction in maxillary arch width in line with previous studies [3, 20]. More strikingly, μCT analysis revealed substantial deterioration in bone quality, with bone volume reduced by nearly 20%, trabecular thickness decreased by 29%, and the BV/TV ratio lowered by 26% compared with the control group. These differences are similar to previous studies [21], and consistent with the decrease in chondrocyte differentiation and transdifferentiation rates. Together, these findings establish a strong mechanistic connection between dietary loading and maxillary development, indicating that diminished functional loading profoundly suppresses chondrocyte‐driven osteogenesis and compromises maxillary bone formation.

The findings of this study parallel our earlier observations in postnatal TMJ condylar growth, in which the decrease in dietary loading altered the extent of condylar growth and remodelling by inhibiting condylar chondrogenesis and chondrocyte transdifferentiation [19]. However, there are several unique features in the mid‐palatal suture compared to the condyle. First, condyle chondrocytes exhibit much higher proliferation activity. There were approximately 150 EdU^+^ cells/mm^2^ in the hard diet condyle, which was seven times higher than that in the mid‐palatal suture. This indicates that even under normal conditions, chondrocytes in the mid‐palatal suture do not replicate nearly as frequently compared to those in the condyle. Second, both the condyle and mid‐palatal sutures showed significant reductions in chondrocyte‐derived osteogenesis in soft diet animals, with no significant differences in non‐chondrocyte‐derived bone formation between groups. However, approximately 70%–80% of bone cells in the superior condylar process of hard diet mice were chondrocyte‐derived, compared to 40%–50% in the mid‐palatal bone area.

One possible factor that may contribute to the lower rate of chondrocyte transdifferentiation in the mid‐palatal suture compared to the condyle is the difference in loading at the two sites. During mastication, the majority of the force in the mandible is distributed at the superior portion of the condyle [22]. Up to 35 pounds of force can be transmitted to the condyle during mastication in monkeys [23]. In contrast, force distributed in the maxilla is mainly received by the zygomatic arch, infraorbital rim and alveolar processes rather than the mid‐palatal suture [24]. The mid‐zygomatic arch receives approximately eight times more of the maximum shear strain than the mid‐palatal suture during mastication [25]. Such large differences in force during masticatory activity between the two regions could lead to less chondrocyte transdifferentiation in the mid‐palatal suture than in the condylar cartilage.

In summary, the present study demonstrated for the first time that chondrocytes in the mid‐palatal suture directly contribute to normal maxillary growth, contrary to the common concept that maxillary development occurs solely through intramembranous ossification [26, 27]. The presence of a large number of hypertrophic chondrocytes within the suture and their cell fate transition during normal growth shows that, in mice, maxillary transverse development is at least partially performed through endochondral ossification. Moreover, reduced dietary loading impaired chondrocyte differentiation and maturation within the suture, resulting in a diminished pool of chondrocytes available for transdifferentiation and consequently restricted maxillary transverse growth (Figure 6). Future functional studies, such as conditional deletion of Acan^+^ cells, mechanical rescue experiments, or maxillary expansion models, will be required to definitively determine the biological functions of chondrocytes in the mid‐palatal suture during development and orthopaedic treatment.

It is worth noting that the mid‐palatal sutures of murine animals differ from those of humans [28]. In mice, secondary cartilage within the mid‐palatal suture persists from embryonic through advanced postnatal ages [29, 30]. In contrast, in humans, cartilage is generally considered to be only transiently present within the mid‐palatal suture during foetal and early neonatal periods and is not regarded as a permanent or defining feature of the postnatal suture during childhood or adolescence [28]. Therefore, it is essential to consider these species‐specific differences when interpreting and discussing the present findings.

Hyperdivergent patients represent one of the most difficult orthodontic populations to manage. Functionally, they exhibit weak masticatory musculature, reduced bite force, and poor chewing efficiency, often accompanied by a narrow maxilla, vertically directed mandibular growth, and smaller muscle fibre size and activity [3]. These phenotypes usually do not self‐correct and typically worsen over time [31]. Previous studies have demonstrated that increased masticatory activity stimulates sutural osteogenesis and enhances maxillary transverse growth [32]. The present study introduces a new therapeutic perspective that strategically enhancing muscle activity or dietary loading could serve as a biologically based approach to stimulate mid‐palatal bone formation and address maxillary constriction in hyperdivergent patients.

On the other hand, the direct contribution from the chondrocytes in the mid‐palatal suture to maxillary transverse growth holds important implications for rapid palatal expansion. Understanding the mechanism in which the mid‐palatal suture matures at a cellular level is the first step in finding future targets for speeding up bone modelling and remodelling during orthopaedic expansion, stabilisation and post‐treatment retention.

## 5 Conclusion

After 6 weeks of reduced dietary loading, mice exhibited structural and cellular changes in the mid‐palatal region:

1. Less chondrocyte differentiation and maturation within the mid‐palatal suture;
2. Fewer chondrocyte‐derived bone cells and total bone cells within the subchondral bone in the mid‐palatal suture;
3. Narrower maxillary arch with reduced bone quality in the bone around the suture.

## Author Contributions

M.S. was responsible for data curation, formal analysis, methodology development and drafting the original manuscript. P.D. and Y.L. contributed to data curation and formal analysis. L.R. supervised the project and provided administrative oversight and validation of findings. M.J.K. contributed to project administration, supervision, validation of findings and drafting the original manuscript. Y.J. was responsible for study conceptualization, project supervision, funding acquisition, overall study coordination, drafting the original manuscript and critical revision of the manuscript.

## Funding

This study is partially supported by National Institutes of Health grants DE028547, DE028593 and DE029541 to Yan Jing; American Association of Orthodontists Foundation‐Biomedical Research Award to Yan Jing.

## Ethics Statement

This study was ethically approved by the Institutional Animal Care and Use Committee of Texas A&M College of Dentistry (IACUC 2022‐0124‐CD).

## Consent

The authors have nothing to report.

## Conflicts of Interest

The authors declare no conflicts of interest.

## Supporting information


**Figure S1:** Experimental design and study workflow. (A) Two compound mouse lines were utilised to trace chondrocyte lineage: Aggrecan‐Cre^ERT2^; R26R^tdTomato^; 2.3Col1a1‐GFP and Col10a1‐Cre; R26R^tdTomato^; 2.3Col1a1‐GFP. Soft diet feeding was initiated at 3 weeks of age to reduce masticatory loading, and all mice were harvested at 9 weeks. A single tamoxifen injection was administered to the Aggrecan‐Cre^ERT2^ mice at 3 weeks to induce lineage labelling. (B) For 3D μCT analyses, the region of interest (ROI) was defined as the maxillary bone between the palatal roots of the first and second molars, extending 100 μm lateral to the mid‐palatal suture. (C, D) Mouse body weights were monitored weekly (C) to assess general health and growth. No significant differences were observed in weight changes between the soft and hard diet groups throughout the experimental period (D). *p < 0.05.

**Figure S2:** Soft diet impairs chondrocyte‐derived bone formation in Col10a1^Lineage^ mice. (A) In hard diet mice, confocal imaging revealed a dense population of Col10a1^Lineage^‐derived bone cells (yellow or red) within the maxillary bone adjacent to the mid‐palatal suture, whereas the soft diet group exhibited a dramatic reduction in these cells. (B) Quantitative measurements corroborated this observation. BC, bone cells. White dotted lines mark the cartilage–trabecular bone interface; white solid lines denote regions approximately 100 μm lateral to the suture boundary.

## Data Availability

The data that supports the findings of this study are available in the [Link], [Link] of this article.
